# Self-Medication Practice and Associated Factors Among Pregnant Women Residing in Kattankulathur Block of Chengalpattu District in Tamil Nadu, India: A Cross-Sectional Study

**DOI:** 10.7759/cureus.67574

**Published:** 2024-08-23

**Authors:** Ezhil Muthalvan, Kaveri P, Logaraj M, Anantharaman VV

**Affiliations:** 1 Community Medicine, SRM Medical College Hospital and Research Centre, SRM Institute of Science and Technology (SRMIST), Chengalpattu, IND

**Keywords:** allopathic medicines, determinants, antenatal care, pregnancy, over-the-counter drugs, self-medication

## Abstract

Background

Self-medication is a prevalent phenomenon worldwide. The self-medication practiced by pregnant women, particularly during the first trimester, can have significant adverse consequences for both the developing fetus and the mother. Although self-medication during pregnancy has the potential to cause adverse effects, there is less information regarding the consequences among pregnant women in low-middle-income countries, such as India. Multiple factors influence the self-treatment pattern and exhibit variation across different societies. This study assessed the self-medication practices (SMPs) of pregnant women living in the Kattankulathur block of Tamil Nadu and identified the socio-demographic factors that are associated with this practice.

Materials and methods

A cross-sectional study was carried out among 403 pregnant women living in Kattankulathur block of Chengalpattu district, Tamil Nadu, India. The pregnant women who accessed antenatal care in the health center under Kattankulathur block were included in the study. The data were gathered via in-person interviews with specifically chosen pregnant women via a semi-structured questionnaire administered by the main researcher. The interview schedule comprised three components. The first section encompasses demographic details, the second part includes parity and stage of the current pregnancy, and the third part covers SMPs.

Results

The study encompassed 403 participants, with a mean age of 27.34 years. Self-medication was reported by 216 participants, which accounted for 53.6% of the sample. Out of the 216 individuals who reported SMPs, 52.8% (114 participants) were primiparous and 47.2% (102 participants) were multiparous, depending on the distribution based on parity. Approximately 32.9% of the participants (71 individuals) were in the first trimester. The second trimester accounted for 35.6% of the participants (77 individuals). Out of the total participants, the largest category comprises professionals, with 167 individuals, accounting for 41.4% of the total. Based on the modified BG Prasad scale, the middle class was the most common group, accounting for 46.4% (187 participants). Approximately 30.6% of the respondents (66 participants) indicated that the rates charged for doctor consultations were huge. Approximately 34.7% of the respondents (75 participants) cited the convenient accessibility of drugs as a contributing factor. The most prevalent problem addressed was headaches, which made up 36.57% (79 participants). Allopathic medicines were chosen by the majority, including 69.0% (149 participants), whereas Ayurvedic medicines were chosen by 22.2% (48 people). The primary sources of medications were expired prescriptions from physicians (32.4%, 70 participants) and suggestions provided by pharmacists (31.0%, 67 participants). Pregnant women who have a higher socioeconomic status, higher academic achievement, and a greater number of family members are engaging in self-medication more frequently.

Conclusion

The prevalence of self-medication among pregnant women in this study was significantly higher compared to the other literature in India. This issue poses a public health concern that has the potential to negatively impact the well-being of both mothers and their unborn children. It is imperative to establish stringent regulations and oversight to ensure responsible SMPs, with the active participation of healthcare professionals and lawmakers.

## Introduction

Currently, the widespread use of medications and the practice of self-medication are recognized as significant health and socioeconomic issues in various countries [1]. The incidence of this condition varies from 11.7% to 92% worldwide [2]. Self-medication refers to the practice of using medications to address disorders or symptoms that individuals have diagnosed themselves or the regular or occasional use of a prescribed substance for chronic or recurring diseases or symptoms [3].

The World Health Organization (WHO) advocates promoting the use of self-medication without medical consultations to get efficient and rapid symptom alleviation, hence easing the strain on healthcare facilities that are frequently understaffed and difficult to access in rural and isolated regions [4]. Over-the-counter (OTC) medications are a type of self-administered medication. The purchaser self-diagnoses their own ailment and buys a particular medication to remedy it [5]. OTC drugs offer alleviation for symptoms of diseases that do not necessitate medical intervention. Despite the indisputable advantages derived from OTC pharmaceuticals, self-medication can have a range of adverse effects, including treatment failures, drug toxicity, increased treatment expenses, prolonged hospital stays, and heightened morbidity rates [6].

Several factors contribute to the rising prevalence of self-medication, including the desire for self-care, empathy toward family members during illness, limited access to medical care, impoverishment, lack of knowledge, misconceptions, aggressive drug advertising, and the availability of drugs outside of pharmacies [7]. Many countries are currently experiencing significant health concerns because of the ongoing global economic slump and the difficulties in meeting the medical demands of their populations. In low-middle-income countries, the prevalence of such a situation often prompts many individuals to engage in self-medication, utilizing various substances and plants to address their medical requirements. The lack of understanding regarding the pharmacological properties of medications and their potential effects on individuals who engage in self-medication is a major factor that amplifies the risks associated with self-medication in developing nations [8,9].

The practice of self-treatment differs across many groups and is influenced by multiple factors including age, gender, income, expenditures, inclination toward self-care, education, medical expertise, satisfaction, and individuals' perception of illness [10]. Many impoverished nations with inadequate healthcare systems often experience a significant prevalence of self-medication among pregnant women [11].

The administration of medication during pregnancy continues to be a medical issue [12]. Despite the common use of medications during pregnancy in clinical settings, their safety has not been thoroughly proven because of the exclusion of pregnant women from scientific trials, which is motivated by concerns about potential harm to both the mother and the developing fetus [12].

Approximately 10% or greater than 10% of birth abnormalities are attributed to the prenatal exposure of pregnant mothers to drugs [13]. Multiple studies have shown that the utilization of drugs and self-administration of medication while pregnant can impact the health of the developing fetus [14]. Unsupervised use of medication by pregnant women, particularly during the initial three months of pregnancy, can cause significant adverse effects on both the developing fetus and the mother. Possible consequences may encompass children's deformity, hindered normal growth of the newborn, impaired maturation of reproductive organs, urine retention, intersex conditions, undescended testis, and other urethral-related issues [15,16].

Pregnant women commonly engage in self-medication for symptoms such as the common cold, headache, anemia, and nausea/vomiting. Women commonly have apprehension regarding the utilization of allopathic or natural drugs while pregnant [6]. Using herbal medicines for self-medication by pregnant women is a matter of significant concern, particularly when considering the cultural significance, accessibility, and cost of traditional cures in severe economic crises [8].

Although self-medication during pregnancy has the potential to cause harm, there is a lack of information regarding its impact on pregnant women in underdeveloped countries, such as India [4,7,17]. Therefore, assessing the practice of self-medication and related factors will offer valuable insights to health policymakers and other stakeholders. This knowledge may plan effective measures aimed at mitigating the potential hazards associated with self-medication during pregnancy. While there have been some studies on self-medication in the general population, [2,18] there is a lack of research specifically on self-medication habits during pregnancy in our context. Therefore, the purpose of this cross-sectional study was to investigate the prevalence of self-medication during pregnancy and its socio-demographic factors.

## Materials and methods

Study settings

This analytical cross-sectional study was carried out in the households of Kattankulathur block in Chengalpattu district, Tamil Nadu. The study took place between March 2023 and March 2024.

Study population

All pregnant women residing in Kattankulathur block of Chengalpattu district, Tamil Nadu, India, were included in the study.

Sample size calculation and sampling method

The prevalence of self-medication among 244 pregnant women in the institution-based cross-sectional survey conducted by Jambo et al. in 2017 was found to be 69.4% [19]. The sample size was derived using the formula N = 3.84 * p * q / d^2^, where p represents the prevalence rate, q represents the complement of p, and d represents the accuracy with a 5% absolute error. The formula yielded a sample number of 329. To account for the non-response rate, a 20% increase was applied to the sample, resulting in a minimum requirement of 396 samples for the study. All registered antenatal women who met the specific eligibility requirements and showed their willingness to take part in the research were eligible for participation.

Inclusion criteria

We have included pregnant women who received antenatal care in the primary health center under Kattankulathur block of Chengalpattu district. The study comprised one participant per family. If there were multiple individuals above the age of 18 in the household during the survey, the oldest individual was questioned.

Exclusion criteria

Pregnant women who were mentally ill and unable to hear and speak during data collection were excluded from the study. Pregnant women who declined participation or did not provide consent, as well as those who could not be reached after three attempts, were excluded from the study.

Data collection procedure

The lead investigator acquired data by conducting face-to-face interviews with pregnant women, using a semi-structured questionnaire. The questions were created following a thorough examination of the existing literature on the subject. It was tested on a subset of 5% of the participants and necessary adjustments were made accordingly. The interview schedule comprised three parts. The primary section encompasses demographic attributes such as age, education, occupation, socioeconomic status, family type, and family size. The second part includes information on parity and the stage of the current pregnancy. The third part explored self-medication practice, encompassing details such as the system of medicines used, the reasons behind self-medication, the diseases for which medication was taken, and the various sources of information used.

Ethics approval

Data collection was conducted after obtaining approval from the Scientific and Ethical Committee of the SRM Medical College Hospital and Research Centre (clearance number: SRMIEC-ST0323-501). The study benefits and methods were communicated to all participants in the local language. Study subjects provided written informed consent.

Statistical analysis

All data were entered in Excel (Microsoft® Corp., Redmond, WA) and analyzed in SPSS Statistics version 26.0 (IBM Corp., released 2019, IBM SPSS Statistics for Windows, Armonk, NY: IBM Corp). Frequency analysis and percentage analysis were used to describe the data using descriptive statistics for discrete variables. When working with continuous variables, the mean and standard deviation were employed. To characterize the data in inferential statistics, statistically significant differences between discrete variables in the two groups were examined using the Chi-square test or Fisher's exact test. The statistical procedures all used a significant level of 0.05 for the probability value.

## Results

The study involved 403 participants with an average age of 27.34 years with a standard deviation of 3.92 years. Out of the participants, 68.5% (n = 276) had families comprising three to five members. A total of 386 participants (95.8%) were Hindu by religion. Among the study participants, the majority belonged to nuclear families (251 participants, 62.3%). Joint families accounted for 37.2% (150 participants), while three-generation families were the least represented, making up only 0.5% (two participants). The largest group of pregnant women were professionals (167 participants, 41.4%), and high school graduates comprised 28.0% (113 participants) regarding their education. The majority of the samples were non-working women (331 participants, 82.1%). The socioeconomic classes were assessed using the modified BG Prasad scale. The largest group was Class 2, comprising 46.4% (187 participants). Class 3 comprised 27.3% (110 participants), while Class 1 included 18.1% (73 participants). The smallest group was Class 4, accounting for 8.2% (33 participants). The basic characteristics of the pregnant women included in this study are shown in Table 1.

**Table 1:** Basic characteristics of the pregnant women.

Sociodemographic variables		Frequency	Percentage
Age in years (mean ± S.D)		27.37 ± 3.91	
Religion	Hindu	386	95.8
	Christian	9	2.2
	Muslim	8	2
Type of family	Nuclear family	251	62.3
	Joint family	150	37.2
	Three generation family	2	0.5
Total number of family members	<3	117	29
	3 to 5	276	68.5
	> 5	10	2.5
Education	Professional	169	41.9
	College	46	11.4
	High school	113	28
	Middle school	30	7.5
	Primary school	45	11.2
Occupation	Working	72	17.9
	Non-working	331	82.1
Socioeconomic status	Class 1	73	18.1
	Class 2	187	46.4
	Class 3	110	27.3
	Class 4	33	8.2

**Figure 1:**
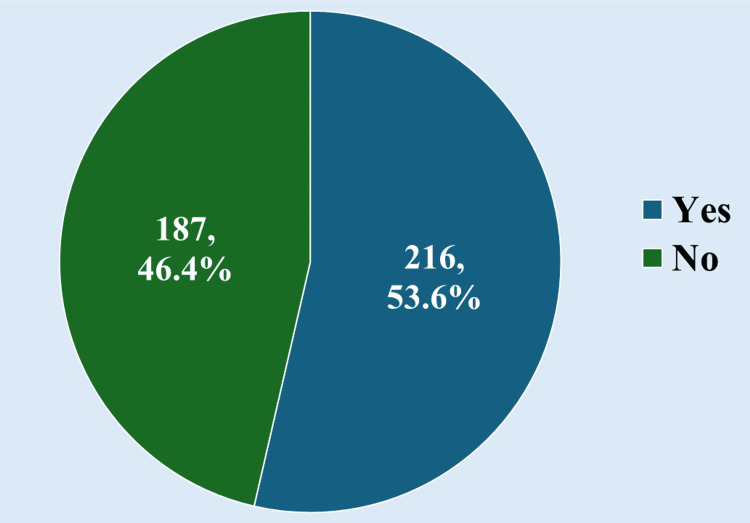
Prevalence of self-medication practice (n = 403)

The study involved 403 participants, among whom 53.6% (216 participants) were taking self-medication. None of the study participants had experienced adverse events with self-medication. Figure 1 shows the prevalence of self-medication practices.

Among the 216 participants, the distribution according to parity shows that 52.8% (114 participants) were categorized as primiparous, while 47.2% (102 participants) fell into the multiparous category. The distribution of antenatal women by trimester was as follows: 32.9% (71 participants) in the first trimester, 35.6% (77 participants) in the second trimester, and 31.5% (68 participants) in the third trimester. Table 2 shows the distribution of the study participants according to the parity and the period of trimester.

**Table 2:** Distribution of the study participants according to the number of parity and the period of trimester (n = 216)

Variables	Frequency (n = 216)	Percent
Number of parities		
Multiparous	102	47.2
Primiparous	114	52.8
Currently under which stage of pregnancy		
First trimester	71	32.9
Second trimester	77	35.6
Third trimester	68	31.5

Among the 216 participants who practiced self-medication, the majority opted for allopathic medicines, accounting for 69.0% (149 participants). Ayurvedic medicines were chosen by 22.2% (48 participants), while homeopathic medicines were selected by 8.8% (19 participants). This distribution shows a predominant preference for allopathic medicines. The most common sources were old prescriptions from doctors (32.4%, 70 participants) and recommendations from pharmacists (31.0%, 67 participants). Previous personal experience accounted for 18.1% (39 participants), while recommendations from peers, friends, or family made up 10.6% (23 participants). Among the 216 participants who reported taking self-medication for various conditions in the last year, headaches were the most commonly treated problem, accounting for 36.57% (79 participants). Other common conditions included running nose (14.81%, 32 participants), fever (18.06%, 39 participants), and cough (11.57%, 25 participants). Table 3 shows the self-medication practices of pregnant women.

**Table 3:** Most frequent ailments, system of medicine, and sources of obtaining information for treatments in self-medication in pregnant women. * Multiple answers

Self-medication practice	Frequency (n = 216)	Percent
System of medicine selected for self-medication		
Allopathic	149	69
Ayurvedic	48	22.2
Homeopathic	19	8.8
How do you know the name of the drugs for self-medication?		
Advertisement	17	7.9
My previous experience	39	18.1
Old prescription of doctor	70	32.4
Recommended by pharmacist	67	31
Used by peers, friends, or family	23	10.6
For which disease you have taken self-medication in the last one year*		
Acidity	10	4.63
Diarrhea	12	5.56
Running nose	32	14.81
Ear pain	2	0.93
Cough	25	11.57
Pain in joints	5	2.31
Vomiting and nausea	17	7.87
Muscle pain	8	3.7
Fever	39	18.06
Dandruff	8	3.7
Dental pain	6	2.78
Faints	21	9.72
Dysentery	3	1.39
Hair fall	8	3.7
Headache	79	36.57
Difficulty in swallowing	3	1.39
Mouth ulcer	6	2.78
Rash	1	0.46
Wounds	3	1.39

**Figure 2:**
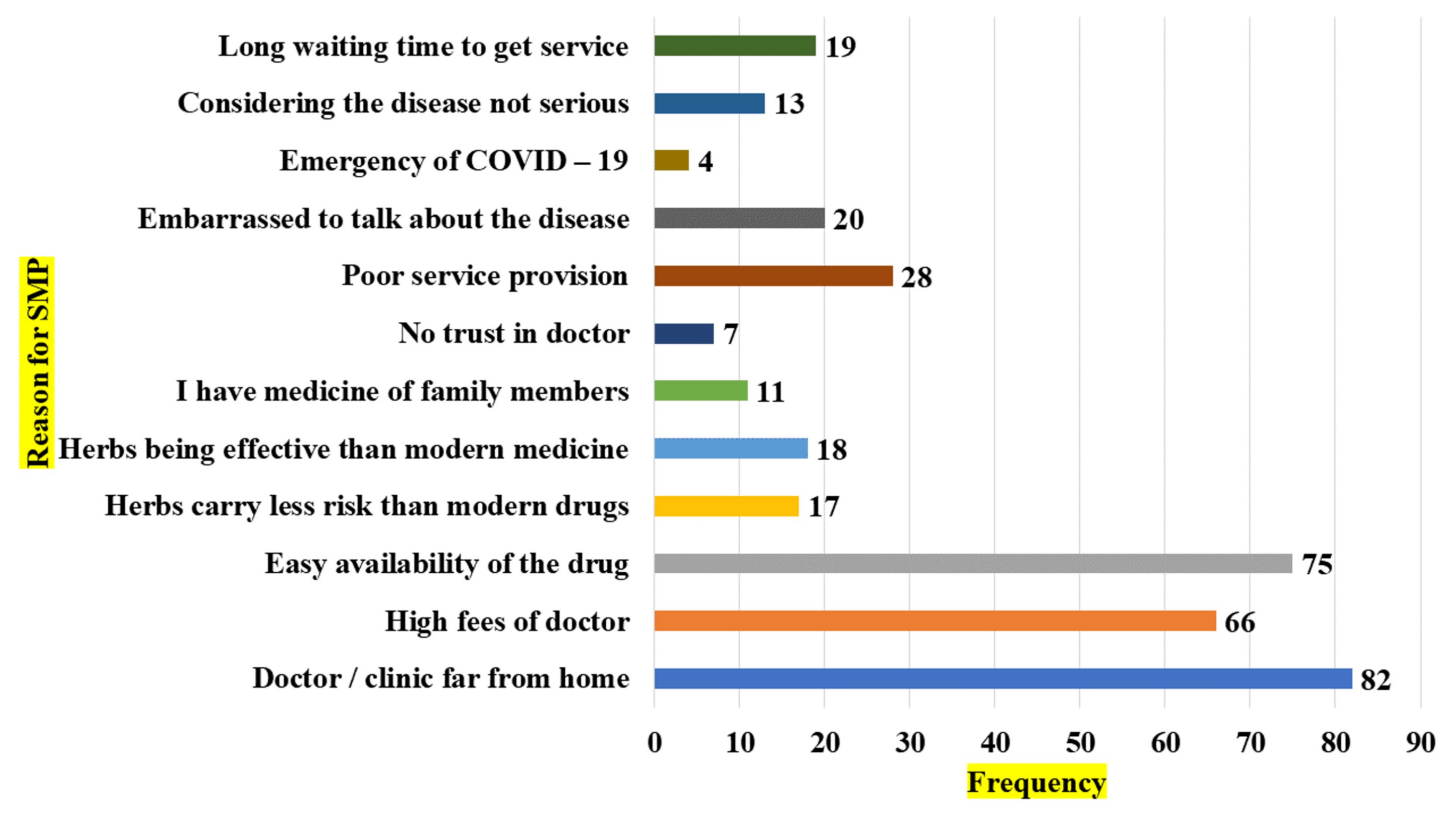
Bar diagram showing reasons for self-medication * Multiple answers

The reasons for self-medication by the study respondents are shown in Figure 2. A significant proportion, nearly 38%, of the pregnant women (82 participants) interviewed reported that the clinic was far from their place of residence. Approximately 30.6% (66 participants) of the sampled individuals perceived the consulting fees as huge, while 34.7%(75 participants) of the respondents acknowledged the easy availability of drugs for purchase.

About 55.6% of the pregnant women and those who were Christian by religion (five participants) practiced self-medication, 53.1% of the samples who were Hindu by religion practiced self-medication (205 participants), and about 75% of the samples who were Muslim by religion practiced self-medication (six participants). Fisher’s exact test shows that there was no relationship between religion and self-medication practice (p-value 0.515).

About 55.3% of the pregnant women (83 participants) and those who were living as a joint family practiced self-medication, 52.2% of the samples (131 participants) who were living as a nuclear family practiced self-medication, and about 100% of the samples who were living as a three-generation family practiced self-medication (two participants). Fisher’s exact test shows that there was no relationship between the type of family and self-medication practice (p-value 0.495).

According to the results of Fisher's exact test, there is a significant association between the number of family members and the rate of self-medication among pregnant women (p-value 0.011). When the family members are five and above, there was a higher chance of self-medication as noticed in the table (100%, n = 10). When the pregnant women completed college studies, there was a higher chance of self-medication (60%, n = 129) compared to women with completed school education, and this difference was statistically significant by the chi-square test (p-value 0.005).

Working pregnant women practiced self-medication (55.5%, n = 40) more than non-working women, and this difference was not statistically significant by the chi-square test (p-value 0.713). Fisher’s exact test shows that as the family income increases, there is a higher chance of self-medication among pregnant women (p-value 0.0001). Table 4 shows the association between the sociodemographic variables and self-medication practice among the pregnant women.

**Table 4:** Association between the sociodemographic variables and self-medication practice among the pregnant women *Fisher's exact test was used and significant at p-value < 0.05.

Self-medication practice versus sociodemographic variables			Self-medication practice		Total (N = 403)	Fisher's exact test value	P-value
			Yes (n = 216)	No (n = 187)			
Religion	Christian	Count	5	4	9	1.466	0.515
		%	55.60%	44.40%	100.00%		
	Hindu	Count	205	181	386		
		%	53.10%	46.90%	100.00%		
	Muslim	Count	6	2	8		
		%	75.00%	25.00%	100.00%		
Type of family	Three-generation family	Count	2	0	2	1.738	0.495
		%	100.00%	0.00%	100.00%		
	Joint family	Count	83	67	150		
		%	55.30%	44.70%	100.00%		
	Nuclear family	Count	131	120	251		
		%	52.20%	47.80%	100.00%		
Total number of family members	<3	Count	63	54	117	9.014	0.011*
		%	53.80%	46.20%	100.00%		
	3 to 5	Count	143	133	276		
		%	51.80%	48.20%	100.00%		
	> 5	Count	10	0	10		
		%	100.00%	0.00%	100.00%		
Education	Completed schooling	Count	87	101	188	7.595*	0.005*
		%	46.20%	53.80%	100.00%		
	Graduated	Count	129	86	215		
		%	60.00%	40.00%	100.00%		
Employment	Working	Count	40	32	72	0.1351*	0.713
		%	55.50%	44.50%	100.00%		
	Non-working	Count	176	155	331		
		%	53.20%	46.80%	100.00%		
Socioeconomic class	Class 1	Count	53	20	73	20.889	0.0001*
		%	72.60%	27.40%	100.00%		
	Class 2	Count	105	82	187		
		%	56.10%	43.90%	100.00%		
	Class 3	Count	45	65	110		
		%	40.90%	59.10%	100.00%		
	Class 4	Count	13	20	33		
		%	39.40%	60.60%	100.00%		

## Discussion

Prevalence of self-medication

The study included 403 participants, of whom 53.6% (216 participants) were engaged in self-medication. No adverse effects were reported among the subjects who self-medicated. Bouqoufi et al. did a systematic review and meta-analysis in 2024 to determine the combined prevalence of self-medication and its related factors among pregnant women [20]. The incidence of self-medication among pregnant women was 44.50%. Mutalub et al. conducted a cross-sectional study of 400 pregnant women in Nigeria and found similar results. About 40% of the pregnant women engaged in self-medication during their pregnancy [21]. Rahmani et al. conducted a systematic review and meta-analysis, retrieving 128 studies to determine the incidence of self-medication among pregnant women in Iran. The researchers observed that the overall prevalence of self-medication was 38.46% [22].

In the study conducted by Chergaoui et al., out of 364 pregnant women, 118 (32%) engaged in self-medication, which was lower compared to the current study [23]. Tujuba et al. performed a cross-sectional study on 585 randomly selected pregnant women in Ethiopia. The study found that the prevalence of self-medication among pregnant women was 19.8%, which was significantly lower compared to the current study [24]. Atmadani et al. undertook a cross-sectional survey among 333 pregnant women, of whom 39 (11.7%) reported using OTC medication. This proportion was significantly lower compared to the current study [25]. We may attribute the variation in prevalence to the fact that our study included both allopathic and herbal drugs for self-medication, whereas other investigations only included one of them.

Reasons for self-medication

The primary cause of self-medication was the considerable distance between the individual's residence and the nearest healthcare institution, as reported by 82 individuals, accounting for 28% of the sample. Approximately 30.6% of the respondents showed the rates charged for doctor's consultations were costly. Approximately 34.7% of the respondents cited the medications' wide availability as a contributing factor.

Dare et al.'s cross-sectional survey identified price, convenience, and lack of transportation to health facilities as the primary reasons for self-medication [26]. Niriayo et al. conducted a study in Ethiopia that involved a cohort of 250 pregnant women. The most frequently cited reasons for self-medication practice, as reported by participants, were ease of access to drugs (25.5%), perception of the ailment as minor (21.6%), and timesaving (19.6%) [16].

In a cross-sectional study conducted by Mutalub et al. in Nigeria, similar results were found. Out of the 400 pregnant women surveyed, 65% said that the medications were easily accessible. In addition, they have indicated that the primary factors contributing to self-medication were the affordability of treatment and the perception that the ailment was of little significance [21]. The availability of medicines without a prescription may be attributed to the lack of government regulation in the distribution process and a disconnect between the pharmaceutical industry and the adherence to professional and ethical standards among those who dispense medication.

Obstetric and gynecological characteristics of the respondents

Out of the 216 individuals, 52.8% (114 participants) were primiparous and 47.2% (102 participants) were multiparous, according to the distribution based on parity. The proportion of the current stage of pregnancy among the 105 participants who reported is as follows: 32.9% (71 participants) were in the first stage of pregnancy, 35.6% (77 participants) were in the middle stage of pregnancy, and 31.5% (68 participants) were in the last stage of pregnancy. The proportion of participants across all phases of pregnancy in this subset of the study is rather uniform.

Tujuba et al. performed a cross-sectional investigation on 585 pregnant women in Ethiopia who were recruited randomly [24]. A portion (31%) of the samples, which corresponds to 181 participants, were in the first trimester. In addition, 56.9% of the samples, equivalent to 333 participants, were in the second trimester. Lastly, 12.1% of the samples, totaling 71 participants, were in the third trimester. Compared to the current study, there were a greater number of patients in the second trimester.

Mutalub et al. conducted a cross-sectional study in Nigeria, including 400 pregnant women. The study found contrasting results, with 44% of the women being single parity and the remaining 56% being multiparous [21]. Out of the total samples, 5% (20 participants) were in the first trimester, 26% (104 participants) were in the second trimester, and 69% (276 participants) were in the third trimester. Compared to the current study, there were a greater number of individuals in the second and third trimesters.

Treatment conditions

Out of the 216 participants who engaged in self-medication for different ailments in the last year, headaches were the most prevalent condition, with 36.57% (79 people) seeking treatment for it. Additional prevalent ailments were rhinorrhea (14.81%, 32 individuals), pyrexia (18.06%, 39 participants), and bronchial cough (11.57%, 25 participants).

Chergaoui et al. conducted a cross-sectional study involving 364 pregnant women, in which 32% engaged in self-medication with contemporary medicine. The primary factors contributing to self-medication were nausea and vomiting, accounting for 29.3% of cases, followed by headache at 24%, heartburn at 23.7%, and back and leg pain at 23% [23].

Bobga et al. completed a community-based cross-sectional study in Cameroon involving 500 pregnant women [6]. The most commonly used medications for self-medication among pregnant women were antiemetics (96, 28.48%), analgesics (72, 21.36%), and antacids (58, 17.21%). The primary symptoms for which pregnant women practiced self-medication were fever/headache (72, 21.36%), constipation (63, 18.69%), and nausea/vomiting (58, 17.21%).

Factors associated with self-medication practice during pregnancy

We observed that pregnant women with a higher socioeconomic status, higher educational attainment, and larger family sizes are more likely to engage in self-medication. The educational attainment of respondents is crucial as several research have shown a correlation between education and the likelihood of engaging in self-medication. One explanation for this phenomenon is that individuals who have more knowledge and experience with self-medication to control illness symptoms are more likely to engage in self-medication during pregnancy. In addition, the expedited relief of symptoms may also be linked to the utilization of alternative medication.

Atmadani et al. conducted a cross-sectional survey among 333 pregnant women, of whom 39 (11.7%) reported using OTC medication. This proportion was significantly lower compared to the current study [25]. According to this study, women who possessed a greater level of understanding of OTC medication were more inclined to engage in self-medication.

The current study's findings align with those of the study conducted by Mekuria et al. It was noted that the utilization of herbal medicine when pregnant is a widespread custom and is linked to factors such as place of residence, educational attainment, and monthly income [27]. Aisyah and Sitorus did a study on pregnant women in Central Java in the year 2023. The findings indicated that the gross regional domestic product per capita is a geographical factor that has a substantial impact on the prevalence of self-medication among pregnant women [28]. Tuha et al. completed a cross-sectional study on 223 pregnant women in a facility setting [29]. Previous exposure to the drug and absence of prior abortion linked to self-administration of conventional medicine, together with a college-level education, prior use of herbal medicine, a specific type of herb used, and a distance of 5-10 km were identified as factors that predicted the practice of self-medication with herbal medicines.

A thorough understanding of the frequency of self-medication and its related aspects would empower healthcare providers to educate and advise pregnant women about the potential consequences of self-medication and the use of herbal medicine. The findings are essential for the Ministry of Health to develop and execute strategies to regulate the marketing, distribution, and usage of both conventional and herbal medications. This will optimize the utilization of medication and encourage the effective usage of medicinal treatment.

The government should form a committee to investigate unlicensed pharmacies and chemist shops, halt the illicit distribution of medications, and impose penalties on pharmacists and chemists engaged in such practices. Healthcare practitioners and consumers should recognize the significant social and economic implications of self-medication. An interactive partnership between patients, pharmacists, and physicians can influence responsible self-medication [30].

Limitations

The study's limited sample size hinders the generalizability of its findings. Conducting a multicentric investigation with larger samples could yield more accurate results. The study findings may be influenced by recall bias and social desirability bias in pregnant women, as they were asked to provide responses based on their personal life experiences. The cross-sectional study design prevented the ability to infer or establish a causal-effect link based on the study's findings.

## Conclusions

The prevalence of self-medication among pregnant women in this study was much higher compared to other literature in India, indicating a significant public health concern. The prevalence of self-medication during pregnancy was considerably higher among pregnant women with a higher socioeconomic status, higher educational attainment, and larger household size. By conducting qualitative research, an in-depth comprehension of the frequency of self-medication and its related aspects would enable healthcare providers to effectively educate and advise pregnant women regarding the potential consequences of self-medication and the use of herbal medicine. These findings are essential for the Ministry of Health to develop and execute strategies to regulate the marketing, distribution, and usage of both conventional and herbal medications. It is crucial to implement strict laws and regulations to ensure responsible self-medication practices, which should include healthcare professionals and lawmakers. Similarly, it is imperative to give priority to extensive public health awareness efforts on all platforms, establish efficient monitoring of medication distribution, and enforce decisive legal actions against medical malpractice.
